# The Sternal Management Accelerated Recovery Trial (S.M.A.R.T) – standard restrictive versus an intervention of modified sternal precautions following cardiac surgery via median sternotomy: study protocol for a randomised controlled trial

**DOI:** 10.1186/s13063-017-1974-8

**Published:** 2017-06-23

**Authors:** Md Ali Katijjahbe, Linda Denehy, Catherine L. Granger, Alistair Royse, Colin Royse, Rebecca Bates, Sarah Logie, Sandy Clarke, Doa El-Ansary

**Affiliations:** 1 Department of Physiotherapy, Melbourne School of Health Sciences, The University of Melbourne, Parkville, VIC 3053 Australia; 2 Department of Physiotherapy, Hospital Cancelor Tuaku Mukhriz, Pusat Perubatan University Kebangsaan Malaysia, Kuala Lumpur, 56000 Malaysia; 3 Department of Physiotherapy, The Royal Melbourne Hospital, Parkville, VIC 3050 Australia; 4 Department of Surgery, University of Melbourne, Parkville, VIC 3010 Australia; 5 Department of Surgery, Royal Melbourne Hospital, Parkville, VIC 3050 Australia; 6 Physiotherapy Department, Melbourne Private Hospital, Parkville, VIC 3052 Australia; 7 Statistical Consulting Centre, School of Mathematics and Statistics, The University of Melbourne, Parkville, VIC 3010 Australia

**Keywords:** Randomised controlled trial, Cardiac Surgery, Median Sternotomy, Sternal Precautions, Physiotherapy

## Abstract

**Background:**

The routine implementation of sternal precautions to prevent sternal complications that restrict the use of the upper limbs is currently worldwide practice following a median sternotomy. However, evidence is limited and drawn primarily from cadaver studies and orthopaedic research. Sternal precautions may delay recovery, prolong hospital discharge and be overly restrictive. Recent research has shown that upper limb exercise reduces post-operative sternal pain and results in minimal micromotion between the sternal edges as measured by ultrasound. The aims of this study are to evaluate the effects of modified sternal precautions on physical function, pain, recovery and health-related quality of life after cardiac surgery.

**Methods/design:**

This study is a phase II, double-blind, randomised controlled trial with concealed allocation, blinding of patients and assessors, and intention-to-treat analysis. Patients (n = 72) will be recruited following cardiac surgery via a median sternotomy. Sample size calculations were based on the minimal important difference (two points) for the primary outcome: Short Physical Performance Battery. Thirty-six participants are required per group to counter dropout (20%). All participants will be randomised to receive either standard or modified sternal precautions. The intervention group will receive guidelines encouraging the safe use of the upper limbs. Secondary outcomes are upper limb function, pain, kinesiophobia and health-related quality of life. Descriptive statistics will be used to summarise data. The primary hypothesis will be examined by repeated-measures analysis of variance to evaluate the changes from baseline to 4 weeks post-operatively in the intervention arm compared with the usual-care arm. In all tests to be conducted, a *p* value *<*0.05 (two-tailed) will be considered statistically significant, and confidence intervals will be reported.

**Discussion:**

The Sternal Management Accelerated Recovery Trial (S.M.A.R.T.) is a two-centre randomised controlled trial powered and designed to investigate whether the effects of modifying sternal precautions to include the safe use of the upper limbs and trunk impact patients’ physical function and recovery following cardiac surgery via median sternotomy.

**Trial registration:**

Australian and New Zealand Clinical Trials Registry identifier: ACTRN12615000968572. Registered on 16 September 2015 (prospectively registered).

**Electronic supplementary material:**

The online version of this article (doi:10.1186/s13063-017-1974-8) contains supplementary material, which is available to authorized users.

## Background

Cardiac surgery via a median sternotomy has been performed in over 1 million cases worldwide [1, 2] because it provides the best clinical outcome for patients with multiple-vessel disease and co-morbidities [3–7]. Despite these advantages, the incidence of sternal complications has remained relatively unchanged for the last 2 decades and is reported to be between 0.4% and 8% worldwide [8–11]. Sternal complications include dehiscence, wound infection, sternal instability/non-union and mediastinitis [11]. These complications are associated with significant morbidity and prolonged hospital length of stay, and they contribute to increased health care costs [8, 11, 12].

To prevent sternal complications, the routine implementation of sternal precautions that place restrictions on the use of the upper limbs and trunk commences immediately post-operatively. These precautions are used worldwide, although they are applied for variable periods of time (4 weeks to 3 months) post-operatively [8, 13, 14]. The evidence to support sternal precautions is limited to cadaver and replica bone model studies [15, 16]. In a foundational study, McGregor et al. found that a force of 220 ± 40 N was required to attain 2-mm distraction between sternal edges in the lateral direction, 263 ± 74 N in the anteroposterior direction and 325 ± 30 N in the rostrocaudal direction [16]. This prompted a recommendation to discourage the bilateral use of the upper limbs because this was thought to increase the distractive forces at the sternal edges [16]. From the outset, health care professionals, including surgeons, nurses and physiotherapists, routinely reinforce sternal precautions in their clinical practice. However, a recent study demonstrated that upper limb and trunk tasks cause only minimal micromotion of the sternal edges (<2 mm) as measured by real-time ultrasound, and this was the case for all tasks, including bilateral and unilateral arm elevation [17]. On the basis of these findings, restricting the use of the upper limbs and trunk in an attempt to prevent excessive sternal motion may be overly cautious. Sternal precautions in the form of physical restrictions may delay recovery, prolong return to function and delay hospital discharge, and as such may be overly restrictive [8, 18, 19].

Upper limb and trunk exercises are encouraged as part of post-operative care to promote recovery and return of function [8, 13, 14, 18]. Sturgess et al. found that exercises of the trunk and upper limb significantly reduced sternal pain during the first 6 weeks post-operatively [20]. The prescription of such exercises alongside sternal precautions poses a clinical dilemma because they contradict each other [8, 13, 44]. Further, physical activity and upper limb exercises may be imperative for healing and remodelling of bone, which responds to loading [8, 21].

### Trial objective and hypothesis

The primary aim of this study is to evaluate the effectiveness of a program of modified sternal precautions on physical function compared with standard care sternal precautions following cardiac surgery via a median sternotomy at 4 weeks post-operatively. We hypothesise that those receiving the modified sternal precautions will have improved physical function at 4 weeks post-operatively compared with participants receiving standard care precautions. The secondary aims are (1) to evaluate the effectiveness of modified sternal precautions compared with standard care on sternal pain and discomfort, kinesiophobia and health-related quality of life (HRQoL) at 4 weeks and 3 months post-operatively, as well as on physical function at 3 months post-operatively; (2) to measure participants’ adherence to sternal precautions; and (3) to explore whether demographic factors, co-morbidities and/or pre, peri- and post-operative risk factors are associated with the development of post-sternotomy complications. This will be an exploratory analysis which may identify trends of predictors reported in the literature [11].

## Methods/design

The methods are reported in accordance with the Standard Protocol Items: Recommendations for Interventional Trials (SPIRIT) guidelines for clinical trials [22] (*see* Additional file 1, Table 1) and the Template for Intervention Description and Replication (TIDieR) reporting of interventions [23] (*see* Additional file 2).

**Table 1:** Additional file World Health Organisation trial registration data set for S.M.A.R.T.

Data category	Information
Primary registry and trial identifying number	Australian New Zealand Clinical Trials Registry number: ACTRN12615000968572
Date of registration in primary registry	16 September 2015
Secondary identifying numbers	N/A
Trial protocol version	This is the version 3 of the protocol and was enacted November 2015
Source(s) of monetary or material support	Nil
Primary sponsor	Nil
Secondary sponsor	Nil
Contact for public queries	Professor Alistair Royse
Contact for scientific queries	Dr Catherine Granger
Public title	Sternal Management Accelerated Recovery Trial (S.M.A.R.T.): The efficacy of modified sternal precautions on improving physical function in patients following cardiac surgery via a midline sternal incision.
Scientific title	A randomised controlled trial of the efficacy of modified sternal precautions versus standard care on improving physical function following cardiac surgery via a median sternotomy
Countries of recruitment	Australia
Health condition(s) or problem(s) studied	Cardiac surgery via a median sternotomy
Intervention(s)	Active comparator: Placebo comparator:
Key inclusion and exclusion criteria	Ages eligible for study: ≥18 years Sexes eligible for study: both Accepts health volunteers: No Inclusion criteria: all adults underwent elective cardia surgery involving a median sternotomy Exclusion criteria: 1. Unable to understand verbal instructions in English. 2. Residing outside Melbourne metropolitan area (i.e., 52-km radius).
Study type	Type: Investigator initiated, interventional, non-pharmacological, pragmatic, study Allocation: concealed randomisation Intervention model: parallel assignment Masking: patient and assessor blinded Primary purpose: prevention
Date of first enrolment	16 September 2015
Target sample size	72
Recruitment status	Completed recruitment on 16 November 2016
Primary outcome(s)	Short Physical Performance Battery (SPPB)
Key secondary outcomes	2. Patient-identified cardiac pain using numeric and visual prompts, Short Form McGill Pain Questionnaire 2 (SF-MPQ-2), Functional Difficulties Questionnaire (FDQ), grip strength, Tampa Scale of Kinesiophobia shortened version (TSK-11), Medical Outcomes Study 36-item Short Form Health Survey (SF-36v2), Global Rating of Change Scales

*S.M.A.R.T.* Sternal Management Accelerated Recovery Trial

### Trial design

The Sternal Management Accelerated Recovery Trial (S.M.A.R.T.) is a phase II, prospective, parallel-group, concealed-allocation, randomised (1:1), controlled, patient- and assessor-blinded clinical trial powered for superiority and being conducted at two tertiary hospitals. Participants will be randomised to participate in the trial if they meet the eligibility criteria, give informed consent and have completed baseline measurement testing performed by a blinded assessor in an outpatient setting. Participants will be informed that they will be randomised to receive either standard or modified sternal precautions before hospital discharge and will be allocated to one of two groups: (1) the control group (standard care) or (2) the intervention group (modified sternal precautions). In addition, participants are asked to provide a global rating of change in physical function using a numeric scale (Global Rating of Change Scales [GRC]).

### Trial setting

The trial will be carried out at two tertiary hospitals: Royal Melbourne Hospital (RMH), and Melbourne Private Hospital (MPH), both located in the state of Victoria, Australia. RMH is a government-funded, university-affiliated teaching hospital, and MPH is a private hospital located adjacent to RMH. This study is being conducted at two major metropolitan hospitals (one private and one public), and the findings can be generalised to both private and public health care settings. Most participants recruited will be geographically located in the same precinct, with the same surgical staff seeing the same population catchment area and the only difference being the source of funding and reimbursement for surgery.

Ethics approval for the study was obtained from the Melbourne Health Human Research Ethics Committee in May 2015 (protocol reference 2015.035). The trial is being conducted in accordance with the Declaration of Helsinki and was registered on 16 September 2015 with the Australian and New Zealand Clinical Trials Registry (ACTRN12615000968572).

### Eligibility criteria

Eligible participants following cardiac surgery via median sternotomy at the participating centres will be invited to participate in the study. They will be identified through their admission to the cardiothoracic ward of both the public and private hospitals.

### Inclusion/exclusion criteria

Participants are eligible for the trial if they meet the following criteria:

- Adults undergoing isolated valve, coronary artery bypass graft (CABG) surgery or a combination of both
- Able to provide informed consent
- Aged 18 years and older

Participants are ineligible for the trial if they meet any of the following criteria:

- Insufficient English-language comprehension
- Reside outside the Melbourne metropolitan area (i.e., 52-km radius)

### Recruitment feasibility

We aimed to recruit 72 participants from among a pool of those admitted for surgery at each centre. Annually, approximately 650 sternotomy procedures are performed at RMH and 450 sternotomy procedures are performed at MPH. Therefore, recruitment of 72 participants is estimated to take 12 months with an average recruitment of 6 participants per week.

### Randomisation and allocation

Randomisation will be conducted by an independent person off-site using a computer-generated random 72 sequence numbering system (from 1 to 72) and a 1:1 allocation ratio. Concealment is via sealed, numbered, double-layered, opaque envelopes. Allocation occurs after baseline testing by opening of the next study envelope by a member of the staff of the university department of physiotherapy who is not involved in the study. The staff member then informs the treating physiotherapist of group allocation. The envelopes will be stored locked in a cabinet, and security measures are in place to prevent unblinding. To avoid allocation bias, Steps will be taken to limit authorised personnel (*n* = 2) with access/permission to open the study envelopes.

### Trial intervention

The implementation of the sternal precautions is performed by the same ward physiotherapist according to allocation for both groups. There will be a different physiotherapist for each participating hospital providing the intervention for both groups. Both physiotherapists are senior clinicians with over 5 years of clinical experience in cardiac surgery. Training will be provided by one independent physiotherapist to ensure consistency in each institution. Standard care is consistent across centres.

### Control group (standard care)

**Fig. 1:**
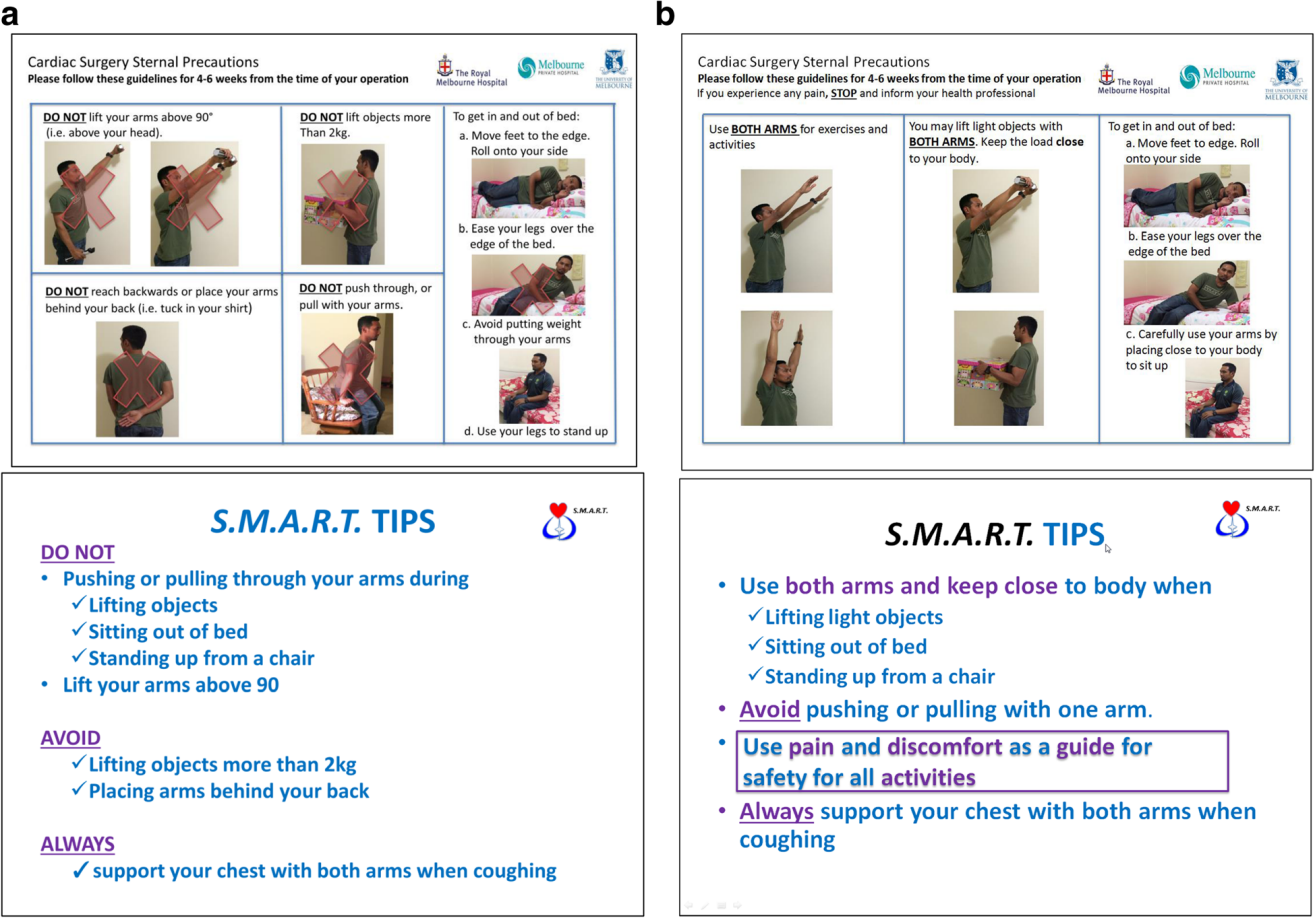
**a** Control group (standard care) participant information flyer. **b** Intervention group (modified sternal precautions) participant information flyer. *S.M.A.R.T.* Sternal Management Accelerated Recovery Trial

Whilst ‘standard care’ is not consistent in the literature cited previously [8, 13, 14, 24, 25], centres worldwide limit the use of the upper limbs for a minimum of 6 weeks [8, 13, 14, 24, 25]. The protocol we will apply is consistent across both institutions in this study. Therefore, consenting participants in the standard care group will receive the education and restrictive sternal precautions for the duration of 6 weeks. The sternal precautions will be delivered in both verbal and written formats by the treating physiotherapists as single individualised sessions for 15 minutes on the ward prior to discharge from the hospital. Patients will be instructed to adhere to the sternal precautions for the first 4–6 weeks post-operatively (Fig. 1a).

The participants will be specifically instructed to do the following:

1. Avoid pushing or pulling through the arms
2. Avoid one-arm (unilateral) activity
3. Limit the elevation of the arms to 90 degrees
4. Avoid lifting objects heavier than 2 kg
5. Use a cushion or perform sternal preservation technique (crossing the arms in a ‘self-hugging’ posture) when coughing
6. Limit the use of the arms when transferring from sitting to standing and getting out of bed
7. Avoid placing the arms behind the back

Participants will be advised not to continue tasks and/or exercises that are painful, to rest as required and to focus on a gradual return to their pre-surgery level of function.

### Intervention group

The modified sternal precautions will be delivered in both verbal and written formats by the treating physiotherapists as single individualised sessions for 15 minutes on the ward prior to discharge from the hospital. Patients will be instructed to adhere to the sternal precautions for the first 4–6 weeks post-operatively (Fig. 1b).

Participants in the intervention group will be specifically encouraged to do the following:

1. Use pain and discomfort to guide the safe use of the arms
2. Avoid pushing or pulling with one arm
3. Use both arms close to the body during lifting
4. Use of arms is possible, but keep them close to the body
5. Avoid stretching one or both arms backwards at the same time
6. Use a cushion or perform sternal preservation technique (crossing the arms in a ‘self-hugging’ posture) when coughing (same as above)
7. When transferring, roll onto the side, ease the legs over the edge of the bed and carefully use the arms to sit up from a lying position

Pain and discomfort should be used to guide the safe limits of movement. The intervention pertaining to sternal management, including the type of sternal precautions, will be delivered in both verbal and written formats to each participant separately with a flyer developed specifically for the study to ensure standardisation. In both groups, all other aspects of patient care, including pre-operative management, general anaesthesia, intra-operative ventilation parameters, fluid delivery, prophylactic antibiotic prescription, pain management, use of lines and drains, general nursing care and discharge planning, will be provided at the discretion of nurses and physicians according to routine clinical practice at both hospitals.

### Intervention fidelity

Training will be provided for the two unblinded, dedicated staff to conduct the follow-up phone calls to ensure consistency in evaluating adherence to sternal precaution guidelines for the first 6 weeks after cardiac surgery. One staff member will evaluate the intervention group and another will follow the standard care group for both institutions. To minimise bias, the staff are required to encourage patients to continue with their allocated sternal management strategy using the standardised written instructions in addition to participants’ flyers. Participants will be informed that they will be contacted via telephone weekly to help reinforce their exercise and precaution guidelines for the first 6 weeks after cardiac surgery. Specifically, the standard care participants will be asked to follow the restriction on the use of their upper limbs and to limit the activities of their upper limbs and trunk during activities of daily living, bed transfers, and sit-to-stand manoeuvers. The intervention group will be encouraged to use their upper limbs bilaterally to perform activities of daily living, bed transfers and sit-to-stand manoeuvers. They will also be encouraged to perform upper limb exercise three times daily within the limits of pain and discomfort.

### Blinding

Patients, outcome assessors and data management are blinded to treatment allocation. Participants will be advised that they will be randomised to one of two groups of sternal precautions guidelines. The treating physiotherapist and nursing staff cannot be blinded to group allocation. The details of sternal management are not documented in the medical record. A blinded assessor located off-site from the hospital will assess all outcomes. Trial staff will conduct education sessions at set times on the ward that are on days separate from days scheduled for outcome assessment. If a treatment group participant informs the assessor of their post-operative education session, this will be noted and reported, and the reason will be entered when the randomisation is unblinded and analysed on an intention-to-treat basis.

### Withdrawal from trial

All participants will be followed after their surgery and measured. Every attempt will be made to accommodate individual requirements to facilitate attendance at follow-up time points beyond discharge from hospital (i.e., taxi vouchers, flexible dates, appointment times). Participants will be withdrawn if they withdraw their consent, and this will be reported. Data collected until this time will be included.

### Data collection

**Fig. 2:**
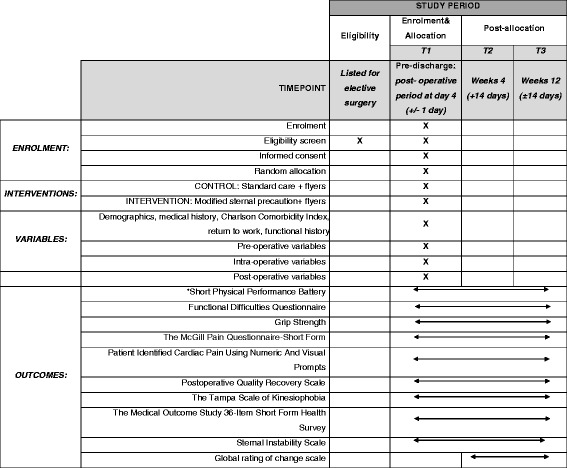
Standard Protocol Items: Recommendations for Interventional Trials (SPIRIT) checklist for the schedule of enrolment, interventions and assessments

Demographic data as well as pre-, intra- and post-operative variables will be collected as listed in Table 2. Data will be collected from the participants and their medical records. All baseline assessments will be performed at the same time of day (08:00–17.00) for each participant in the post-operative period at day 4 (±1 day) in the in-patient setting across centres to minimise potential bias in recruiting participants. The follow-up, outpatient testing at 4 weeks (±14 days) and 3 months (±14 days) will take place in the research room at RMH (Fig. 2). An independent and trained assessor (located off-site) who is blinded to allocation will conduct all measurement sessions. All tests and questionnaires will be administered face-to-face by the outcome assessors and carried out at 4 weeks and 3 months post-operatively to ensure consistency across participants. Post-hospital discharge follow-up will be conducted via phone. If participants are unable to be contacted by phone for a period of 14 consecutive days from the assessment due date, they will be considered lost to follow-up for purposes of post-discharge outcome measurement.

**Table 2:** Data collection details

Demographic data • Name and contact details • Date of birth • Sex • Marital status • Height and weight • Occupation status • Education status • Smoking history • Past medical history and comorbidity index (Charlson comorbidity index) • Functional history (including pre-morbid functional level, use of gait aids, dominant upper limb) • Date of admission to and discharge from acute setting	
Pre-, intra- and post-operative variables • Date of cardiac surgery • Clinical information (left ventricular function, Canadian Cardiovascular Society functional classification, co-morbidities, graft type) • Type of cardiac surgery (including whether it was an emergency or elective procedure) • Other intra-operative details (including method of sternal closure, cardiopulmonary bypass time, operation time, adverse events) • Date of admission to and discharge from intensive care unit post-operatively • Risk factors for pre-, intra- and post-operative (i.e., duration of mechanical ventilation) • Type and use of pain medication (pre- and post-operatively) • Type and use of other medications (pre- and post-operatively) • Date of admission to and discharge from acute physiotherapy services • Details of physiotherapy treatment (including exercises and education provided) • Other adverse events during hospital admission (pre- and post-operatively leading to increase in length of stay); this includes superficial sternal infection, deep sternal infection, re-wiring, re-operation, pneumonia as defined in the Australian Society of Cardiac and Thoracic Surgeons (ASCTS) data • Date of readmission

### Outcomes assessment

#### Primary outcome: Short Physical Performance Battery

The Short Physical Performance Battery (SPPB) is a functional test that measures daily functional activities in the acute care in-patient older population, including cardiac surgery [26]. The test is a well-established and validated measure of lower extremity performance and is designed to simulate routine physical activities in older adults [27]. The test includes gait speed (8-foot walk), standing balance, and lower extremity strength and endurance (chair rise task). It is comprised of the following:

1. *Gait speed:* Participants will be instructed to walk a distance of 8 feet as determined by traffic cones on a flat surface at their normal, comfortable pace. The average of two trials will be used. For safety reasons, participants are encouraged to walk with their gait aids if these are usually used or are part of their post-operative care at the time of testing. An 8-foot course was used, and scoring will use the faster of the two walk times to calculate speed in metres/second. A reduction of the distance to measure gait speed has been shown to provide valid data in measuring of functional limitations [28, 29].
2. *Standing balance:* Participants will be assessed in three different static positions (side-by-side stand, semi-tandem stand and tandem stand). Participants will be instructed to try to hold each of these positions for 10 seconds.
3. *Chair rise task:* Participants will be instructed to stand up and sit down five times in a row as quickly as possible.

The SPPB score is based on timed measures of standing balance, walking speed and ability to rise from a chair. Each test is scored on a scale of 0 to 4 points, with a summary performance score range of 0–12 points using cut-point criteria established by Guralnik et al. [27]. A 0 score indicates poor function, whilst 12 indicates excellent function. If the participant is unable to perform a specific test, a score of 0 will be assigned. A score of 10 or lower is considered the cut-point for mobility impairment [27]. The SPPB was selected because it assesses overall functional performance that reflects physical function required to perform everyday tasks. It is hypothesised that the intervention will impact overall functional performance because this is the primary concern of most patients at medium- to long-term follow-up (6 weeks to 12 months) [26, 30]. The test has established validity and reliability in measuring physical performance in the elderly [27, 31, 32] with an intra-class correlation coefficient (ICC) equal to 0.82 [32], and it is a strong predictor of disability in non-disabled older persons [33]. The minimal detectable change values range from 0.54 [34] to 2 points [35], which suggests that a change in physical performance of 1 to 2 points is a clinically meaningful change in an older [34] and in-patient stable cardiovascular population [35]. Therefore, in the absence of data for cardiac surgery populations, the minimal important difference (MID) for this study was derived from prior research with a cohort of patients with stable cardiovascular conditions of 2 points, which is representative of our participant population. The SPPB has been shown to be reliable, valid and sensitive to change [36]. ICCs ranged from 0.88 to 0.92 for measures done 1 week apart, with a 6-month average ICC of 0.78 [36].

#### Secondary outcomes

1. *Functional Difficulties Questionnaire (FDQ):* The FDQ measures the functional status of patients following cardiac surgery, with a particular focus on upper limb and trunk function in patients following a median sternotomy [37]. The questionnaire requires patients to rate the difficulty they would experience when completing a series of 13 upper limb and trunk functional tasks. Specifically, patients are asked to place a mark along a 10-cm line, with anchors indicating ‘no difficulty’ and ‘maximum difficulty’ on the left and right sides of the line, respectively. For those activities that participants cannot complete while filling out the questionnaire, they will be asked to recall the last time they performed the tasks. The 13 functional tasks included in the questionnaire are everyday tasks that were nominated as difficult to perform in a pilot study of patients following cardiac surgery [37]. Previous research has demonstrated that the FDQ is a valid, reliable and responsive measure in this patient population with minimal recall bias, and it has been used to measure the functional status of patients following cardiac surgery in both the short term (4 weeks post-operatively) and long term (3 months post-operatively) [37]. The follow-up time points are a minimum of 4 weeks to 2 months apart, thus further reducing recall bias.
2. *Patient-identified cardiac pain using numeric and visual prompts:* This is a pain outcome measurement tool that was developed by Teoh et al. [38]. To obtain data regarding symptom presentation, participants are required to identify on a gender-neutral silhouette torso all locations of their pain or discomfort. The participants are also required to identify their ‘chief’ or ‘main’ symptom, describe its nature by pointing to pictorial identifiers that visually represent a description of the pain (i.e., stabbing, heavy, shooting, burning, squeezing) [39]. The intensity of the pain forms the last domain and uses a Likert-type scale. This tool was selected because it evaluates multiple dimensions of pain and discomfort, is easy to administer and accounts for cultural diversity [40].
3. *Short Form McGill Pain Questionnaire 2 (SF-MPQ-2):* Pain quality will be measured using the SF-MPQ-2, which consists of 22 items investigating 4 dimensions of pain quality (continuous, intermittent, neuropathic and affective) on an 11-point numerical rating scale [41]. The total score is calculated from the mean of 22 items, and scores for the 4 dimension subscales are calculated from the mean of the items included in each subscale. Scores on each subscale can range from 0 to 10. A higher score indicates more severe pain [41]. Participants will be instructed to choose the number that best describes their intensity of pain and related symptoms experienced during the past week. A 0 score will be assigned if the word does not describe the participant’s pain or related symptoms. The original version of the scale (SF-MPQ) has well-established reliability in cardiac populations with α coefficients ranging from 0.75 to 0.83 across various post-operative days [42, 43]. The SF-MPQ-2 is sensitive to change in chronic pain, and total and subscale scores are responsive to change. The changes are associated with patient ratings of global improvement in clinical trials [41].
4. *Tampa Scale of Kinesiophobia shortened version (TSK-11):* The TSK-11 is a widely used tool to measure pain-related fear beliefs about movement and re-injury [44]. It is an adaptation of the original 17-item instrument [44] designed to assess fear of movement or re-injury that excludes the 4 original reverse-scored items that were found to have small item-to-total score correlations. The adapted score is an 11-item instrument where respondents will be asked to rate each item on a 4-point Likert scale, ranging from 1 (strongly disagree) to 4 (strongly agree). The TSK-11 is a reliable and valid measure of fear of movement or re-injury in patients with chronic pain [45, 46]. It has internal consistency, reliability and convergent validity with a Cronbach’s α of 0.80 for the total score [46]. A reduction of at least 4 points on the measure maximises the likelihood of correctly identifying an important reduction in fear of movement [46].
5. *Grip strength:* Hand-grip strength will be measured in kilograms with a hand-held Jamar dynamometer (Performance Health, Warrenville, IL, USA). The participant will be tested in the position recommended [47]. The peak value of the maximal squeeze over 5 seconds will be recorded [48]. Time intervals were allowed between tests. A previous study showed similar test-retest reliability with one trial alone, a mean of two or three trials and a maximum of three trials [49]. In addition, because of influences of pain after surgery, the average may not reflect true performance. Therefore, in this study, three serial tests of maximum grip strength with the dominant hand will be performed, and best of the three values will be recorded. Hand-held dynamometry is a reliable, objective tool for muscle strength measurement [50] and a predictor of post-operative complications, mortality and functional decline [51]. The test is a reliable and responsive measure for patients in cardiac rehabilitation (ICC 0.97 for right and left hand grip strength) [52].
6. *Medical Outcomes Study 36-item Short Form Health Survey (SF-36v2):* HRQoL will be evaluated by SF-36v2, which is a generic measure to assess eight domains, including physical functioning, role physical functioning, role emotional functioning, mental health, vitality, social functioning, bodily pain and general health. All scale or single-item measurements range in score from 0 to 100 and will be administered by interview. The raw sub-scale scores will be transformed to ‘norm-based’ scores using published algorithms [53]. Norm-based physical and mental component summary scores will be calculated from raw sub-scale scores, with higher scores indicating better quality of life. A higher score on the SF-36v2 sub-domains represents a high level of functioning and higher quality of life [54]. The scale has good reliability, with Cronbach’s α values ranging from 0.65 to 0.96 for all subscales [54]. The instrument can differentiate between levels of health among post-CABG individuals at a single time point and over time [55]. Furthermore, the SF-36v2 is valid in written format as well as verbal administration over the telephone in cardiac patients [56].
7. *Modified Sternal Instability Scale (SIS):* The modified SIS will be used to assess sternal instability. It is a manual test that measures the stability of the sternum on a 4-point scale (0–3)﻿. A score of 0 corresponds to a clinically stable sternum with no detectable motion or separation of the sternal edges, whilst a score of 3 corresponds to a completely separated sternum with marked increased motion or separation of the sternal edges﻿. The original 5-point (0–4) SIS is a valid and reliable clinical tool for measuring the stability of the sternum in patients following a median sternotomy [8, 57]. It has excellent inter- and intra-rater reliability, with ICCs of 0.97 and 0.98, respectively [58].
8. *Adherence monitoring:* A questionnaire was developed by the researchers to monitor the level of adherence of all patients following cardiac surgery via median sternotomy. The list of activities, duration of adherence and rate of adherence are rated on a numeric scale. Participants are prompted to complete the questionnaire by telephone on a weekly basis. They are additionally encouraged to follow the guidelines on sternal management as per their specific flyers. An adherence threshold for the experimental group was set at a participant self-perceived reported rating ≥70%.

#### Global Rating of Change Scales

The GRC (7-point scale) will be administered to participants prior to performance-based assessment at 4 weeks and repeated at 3 months. Participants will be asked to answer the following question: ‘How does your overall physical function now compare with your physical function just before you went home from the hospital?’ and respond according to a 7-point scale ranging from 1 = very much improved to 7 = very much worse (Tables 3 and 4)*.* It has previously been reported in the literature that in the case where patients rate their change as ‘minimally improved’, ‘no change’ or ‘minimally worse’, it is unlikely that a clinically important difference has occurred [60]. In this case, these responses will be re-defined as ‘unchanged’ [59, 60]. A clinically important difference will be considered to have occurred if patients rate their change as ‘much worse’, ‘very much worse’, ‘much improved’ or ‘very much improved’, and these will be re-defined as ‘changed’ [60].

**Table 3:** Global Rating of Change Scales for overall physical function

How does your overall physical function now compare with your physical function just before you went home from the hospital?	
1. Very much improved 2. Much improved 3. Minimally improved 4. No change 5. Minimally worse 6. Much worse 7. Very much worse

**Table 4:** Global Rating of Change Scales for upper arm and body function

How does your arm and upper body function now compare with your arm and upper body function just before you went home from the hospital?	
1. Very much improved 2. Much improved 3. Minimally improved 4. No change 5. Minimally worse 6. Much worse 7. Very much worse	

### Sample size

Sample size calculations were performed for the primary outcome: SPPB. On the basis of acute care in-patient populations and using the MID between the treated group of 2 points of a total possible score of 12 points, with an SD of 2.7 points [36], it is anticipated that 29 participants are required per group (58 in total), based on a two-sample *t* test. This was based on a type I error rate of 0.05, which is consistent with recommendations and a power of 0.80 [61]. This sample was increased to 72 participants on the basis of a predicted 20% dropout rate, based on our previous study in the same population conducted at both participating hospitals [17].

### Data management and quality

We will use the online REDCap database (https://redcap.healthinformatics.unimelb.edu.au/) supported by the University of Melbourne. High data quality will be aimed for through training of those who collect, check and enter study data as well as by regular data checks for inconsistency between and within measurements and missing data. A check will be performed to evaluate the correctness of the randomisation before the start of the statistical analysis. The data and safety monitoring committee (DSMC), with two independent clinical members and one independent statistician, will act in an advisory capacity for the clinical investigators to monitor withdrawals and review ethical conduct and serious adverse events. Further details will be provided in the DSMC charter, once it is developed.

### Statistical methods

Statistical analyses will be performed by the biostatistician. All data will be analysed using the intention-to-treat principle. Descriptive statistics, including mean and SD, median and interquartile range, number and percent, and frequency will be used to summarise data (depending on distribution and type of data). This also includes participant demographics and adherence to sternal precautions. A comparison between the two hospitals will be conducted on the demographic profile of the participants to establish differences in each presenting population.

The primary outcome—the change from baseline to 4 weeks in the SPPB—will be analysed using a mixed between-and-within subjects analysis of variance with repeated measures across participants. The primary hypothesis will be examined by a contrast evaluating change from baseline to the 4-week time point in the modified sternal precaution group compared with the standard care group. The analysis will be carried out according to the intention-to-treat principle, based on the groups to which participants were randomised. The interactions between group and time will be examined first to assess the effect of intervention, and, if no interaction is present, then group and time main effects will be examined. If there are issues with non-normality or ceiling/floor effects of the SPPB, transformation or dichotomisation will be considered. If there are participants who are not following the assigned group protocol, we will consider a supplementary per-protocol analysis. Key secondary outcome data (including upper limb function, pain, kinesiophobia and HRQoL) will be summarised and analysed similarly to the primary outcome.

Logistic regression will be used to determine pre-, peri- and post-operative risk factors associated with the development of post-sternotomy complications. This will be an exploratory analysis which may identify trends of predictors reported in the literature having an individual effect on post-operative sternal complications (i.e., female sex, diabetes mellitus, obesity, bilateral internal mammary artery grafts, re-operation for post-operative complications, and blood product requirement were reported as significant predictors of sternal infection). For all tests conducted, a *p* value <0.05 (two-tailed) will be considered statistically significant, and mean differences (95% confidence intervals) will be reported.

### Duration and timeline

**Fig. 3:**
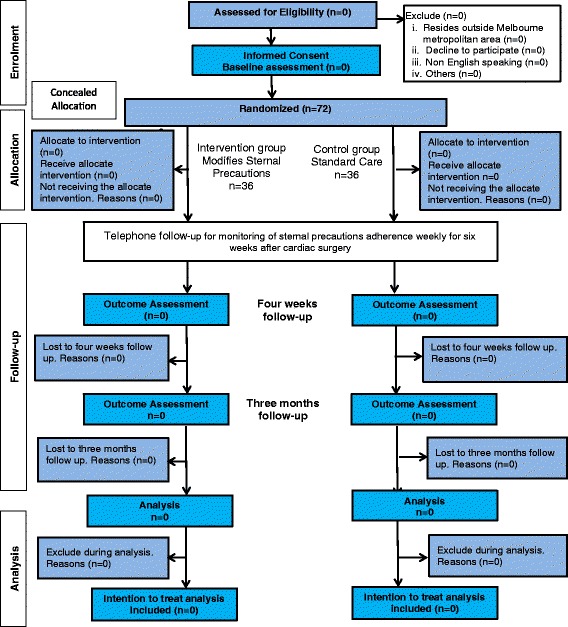
Proposed Sternal Management Accelerated Recovery Trial (S.M.A.R.T.) Consolidated Standards of Reporting Trials (CONSORT) flowchart

The manuscript will be prepared for submission, by July 2017. The final manuscript will be written in accordance with the proposed Consolidated Standards of Reporting Trials (CONSORT) extensions for a pragmatic trial using a non-pharmacological intervention (Fig. 3).

## Discussion

The S.M.A.R.T. study will examine whether modified sternal precautions will facilitate recovery and function following cardiac surgery via a median sternotomy. The benefits of modifying sternal precautions have not been established, despite emerging evidence indicating that a precautionary approach rather than a restrictive approach may be preferable in this patient population [8, 17, 19, 62]. This will be the first randomised controlled trial using an intervention group to modify sternal precautions and to study its effectiveness in improving physical function in this population.

Patients worldwide are currently being prescribed sternal precautions that restrict the use of their upper limbs and trunks to prevent sternal complications for 4–6 weeks [8, 14, 25]. The aims of this restriction are to promote sternal osteosynthesis and bone healing by minimising motion between the sternal edges [8, 15, 63]. However, the effect of sternal precautions on patient outcomes is unknown, with significant variation among institutions worldwide [8, 13, 14, 26]. In addition, there is limited evidence to support their widespread application in clinical practice [8, 13, 15, 18, 62, 64, 65].

Previous studies have shown that unsupported, frequent coughing is the single main cause of mechanical stress through the sternum and may be a far more significant factor in the development of sternal complications [17, 19]. Further, recent evidence demonstrated that upper limbs and trunk movement cause minimal micromotion of the sternal edges (<2 mm) as measured by real-time ultrasound [17]. Therefore, it was proposed that strict post-operative movement restrictions may not be necessary for all patients [8, 13]. However, upper limb movements are part of post-operative standard physiotherapy treatment. In some institutions, this represents instructions on ‘no use of the arms’, or limiting the use of the arms to 90-degree elevation for varying periods of time [8, 13, 14, 25]. Concurrently, patients are encouraged to perform active movements of the upper limbs as part of their post-operative care following cardiac surgery with the aim of restoring physical function [8]. This creates a clinical dilemma collectively for both health professionals and patients [8, 13]. On the basis of findings of a recent survey conducted in Australia [13], we have chosen to modify sternal precaution guidelines encouraging the use of bilateral upper limbs and trunk activities with pain and discomfort as a safety guide in the intervention group to optimise sternal healing and functional recovery in this patient population. Specifically, participants will be allowed to resume their normal load-bearing activities at their own pace within pain-free limits by keeping their upper arms close to their body for common activities (e.g., getting out of bed, lifting and transferring). We hypothesise that this intervention will be safe and cause no harm to the participants. In addition, prior research suggests that unloaded movements within a pain-free range and loaded activity with the upper arms close to the body will not cause excessive stress on the sternal surgical site or bone [8, 19, 62, 65].

Encouraging movement of upper limbs and trunk activities early after cardiac surgery in the post-operative period is recommended in clinical practice worldwide [8, 14, 25] to improve functional outcome [22]. Clinical recommendations will be informed by future analysis of the efficacy of the trial in improving physical function and other associated outcomes. This study will address the paucity of research and the inconsistent recommendations worldwide with respect to sternal precautions and associated restrictions to upper limb and trunk provided to the large number of individuals undergoing cardiac surgery via median sternotomy worldwide. In particular, this research will inform guidelines for the commencement of upper limb exercises in cardiac rehabilitation and standards for sternal precautions and management following cardiac surgery.

### Trial status

All follow-up was completed in April 2017.

## Additional files

Additional file 1: SPIRIT 2013 checklist: recommended items to address in a clinical trial protocol and related documents. (DOC 123 kb)

Additional file 2: The TIDieR checklist: information to include when describing an intervention and the location of the information. (DOCX 33 kb)

## Abbreviations

CABG: Coronary artery bypass graft
CONSORT: Consolidated Standards of Reporting Trials
DSMC: Data and safety monitoring committee
FDQ: Functional Difficulties Questionnaire
GRC: Global Rating of Change Scales
HRQoL: Health-related quality of life
ICC: Intra-class correlation coefficient
MID: Minimal important difference
MPH: Melbourne Private Hospital
RMH: Royal Melbourne Hospital
SF-36v2: Medical Outcomes Study 36-item Short Form Health Survey
SF-MPQ-2: Short Form McGill Pain Questionnaire 2
SIS: Sternal Instability Scale
S.M.A.R.T.: Sternal Management Accelerated Recovery Trial
SPIRIT: Standard Protocol Items: Recommendations for Interventional Trials
SPPB: Short Physical Performance Battery
TIDieR: Template for Intervention Description and Replication
TSK-11: Tampa Scale of Kinesiophobia shortened version
